# Development and validation of a prediction model for primary non-response to IL-17A inhibitors in psoriasis

**DOI:** 10.3389/fmed.2026.1725638

**Published:** 2026-03-25

**Authors:** Qian Yu, Yuxiong Jiang, Yifan Hu, Min Dai, Siqi Li, Jiangluyi Cai, Nan Yang, Yuanwenke Zhang, Yuemeng Huang, Chunyuan Guo, Yuling Shi, Jiajing Lu

**Affiliations:** 1 Department of Dermatology, Shanghai Skin Disease Hospital, Tongji University School of Medicine, Shanghai, China; 2 Institute of Psoriasis, Tongji University School of Medicine, Shanghai, China

**Keywords:** IL-17A inhibitors, prediction model, primary non-response, psoriasis, real-world study

## Abstract

**Background:**

Interleukin-17A (IL-17A) inhibitors have revolutionized the treatment of moderate-to-severe plaque psoriasis, but a substantial proportion of patients experience primary non-response during the early treatment phase.

**Objectives:**

To identify clinical predictors of primary non-response to IL-17A inhibitors and to develop and validate a prediction model for individualized risk assessment.

**Methods:**

A total of 617 patients initiating IL-17A inhibitors in the Shanghai Psoriasis Effectiveness Evaluation Cohort (SPEECH) were randomly allocated into a training set (*n* = 370) and an internal validation set (*n* = 247) in a 6:4 ratio. An external validation cohort included 511 psoriasis patients receiving IL-17A inhibitor across 26 hospitals in China. Primary non-response was defined as failure to achieve ≥75% improvement in the Psoriasis Area and Severity Index (PASI75) at week 12. Logistic regression was used to identify independent predictors, and a multivariable model was developed and validated using receiver operating characteristic (ROC) analysis, calibration curves, and decision curve analysis (DCA).

**Results:**

In the training cohort, 23.2% of patients met the definition of primary non-response. Clinical predictors included higher BMI, absence of family history of psoriasis, prior biologic exposure, and higher baseline PASI score. In addition to these clinical factors, failure to achieve PASI40 at week 4 was a strong indicator of subsequent non-response. The final prediction model exhibited strong discrimination (AUC 0.766, 95% CI, 0.690–0.842), good calibration between predicted and observed probabilities, and meaningful clinical usefulness as demonstrated by DCA. Performance remained robust in both the internal validation cohort (AUC 0.706) and the external validation cohort (AUC 0.708). An online calculator was developed to facilitate individualized risk estimation.

**Conclusion:**

This study identified key baseline and early-response predictors of primary non-response to IL-17A inhibitors and established a validated prediction model with clinical utility. The web-based tool may support precision decision-making in psoriasis management by enabling early identification of high-risk patients.

## Introduction

Psoriasis is a chronic, immune-mediated inflammatory skin disease with a prevalence of approximately 2%–3% worldwide (1). The interleukin-17A (IL-17A) signaling pathway plays a central role in its pathogenesis, driving keratinocyte hyperproliferation and amplifying cutaneous inflammation (2). Targeting this axis has therefore become a key therapeutic strategy. IL-17A inhibitors, such as secukinumab and ixekizumab, have demonstrated rapid onset of action, robust efficacy, and favorable safety profiles in patients with moderate-to-severe plaque psoriasis (3, 4).

Despite these advances, a considerable proportion of patients do not benefit as expected. Some individuals exhibit primary treatment failure, meaning they show little or no clinical improvement during the initial phase of IL-17A inhibitor therapy (5). This phenomenon is of particular concern in clinical practice: patients who fail early lose valuable time in achieving disease control, may face unnecessary costs, and often experience diminished confidence in treatment (6, 7). For clinicians, the unpredictability of primary failure complicates treatment planning and highlights the need for reliable tools to anticipate outcomes.

Accumulating evidence suggests that certain baseline clinical features and early treatment responses may influence the likelihood of therapeutic success with IL-17A inhibitors (8, 9). However, most studies addressing this issue are limited by small sample sizes, heterogeneous designs, or lack of external validation. As a result, there is currently no robust, widely accepted model to help clinicians identify patients at high risk of primary failure in routine practice. In this study, we leveraged two real-world cohorts to systematically investigate the clinical factors and early responses associated with primary treatment failure to IL-17A inhibitors. Based on these findings, we developed and validated a practical prediction model and further implemented it as an online tool to facilitate individualized risk assessment and support precision decision-making in psoriasis care.

## Materials and methods

### Study design and patient population

This study included data from two independent cohorts. The first cohort was derived from the SPEECH cohort (10, 11), a multicenter observational registry study. Eligible participants met the following criteria: (1) adults aged ≥ 18 years; (2) diagnosed with moderate-to-severe plaque psoriasis; and (3) initiation of IL-17A inhibitor therapy. Exclusion criteria included: (1) active infections, other autoimmune diseases, uncontrolled malignancies, or other severe conditions potentially interfering with treatment outcomes; (2) pregnancy or breastfeeding; and (3) absence of available follow-up data.

In total, 617 psoriasis patients who initiated IL-17A inhibitor therapy (secukinumab or ixekizumab) were enrolled and randomly assigned into a training cohort (*n* = 370) and an internal validation cohort (*n* = 247) in a 6:4 ratio. The external validation cohort was obtained from a prospective, multicenter, non-interventional real-world study conducted across 26 hospitals in China between 31 August 2020, and 9 May 2022 (12). Using similar eligibility criteria, 511 patients treated with IL-17A inhibitor were finally included as an external validation cohort. The Clinical Research Ethics Committee of Shanghai Skin Disease Hospital approved the study (approval #2020-36), and all participants gave informed consent.

### Data collection

At baseline, detailed demographic and clinical information was systematically recorded for each patient. Variables included age, sex, body mass index (BMI), family history of psoriasis, smoking status, alcohol consumption, prior biologic treatment, education level and comorbid conditions (obesity, hypertension, and hyperlipidemia). Baseline disease severity was assessed by the Psoriasis Area and Severity Index (PASI). Treatment response was evaluated at baseline, week 4, and week 12. The primary outcome of interest was primary non-response, defined as failure to achieve at least 75% improvement in PASI from baseline after 12 weeks of IL-17A inhibitor therapy.

### Variables analyze and model development

To identify factors associated with primary non-response, baseline profiles were contrasted between responders and non-responders. Continuous data were presented as medians with interquartile ranges (IQR) and assessed using the Wilcoxon rank-sum test. Categorical variables were reported as frequencies with percentages and compared using the Chi-square or Fisher’s exact test, as appropriate. Candidate clinical predictors were first screened through univariable logistic regression analysis, followed by multivariable logistic regression to identify independent predictors. The prognostic value of early PASI improvement at week 4, defined as the percentage reduction in PASI score from baseline to week 4, was first evaluated as a continuous variable using receiver operating characteristic (ROC) analysis to predict primary non-response at week 12. The Youden Index (YI), defined as sensitivity + specificity−1, was applied to determine the optimal cutoff across PASI thresholds. Additional correlations were explored using Spearman’s rank test, with statistical significance set at *p* < 0.05.

### Model assessment

The selected variables were incorporated into a predictive model and visualized as a nomogram. Model performance was evaluated by the area under the ROC curve (AUC) for discrimination. Model calibration was assessed using bootstrap-corrected calibration curves, which reflect overall prediction error (Brier score), systematic over- or underestimation of risk (calibration intercept), and potential overfitting or underfitting (calibration slope). Clinical utility was examined through decision curve analysis (DCA) to estimate net benefit across varying thresholds. All analyses were conducted in R (version 4.2.1) using the pROC package for ROC curves, rms for calibration and nomogram construction, and rmda for DCA.

## Results

### Baseline characteristics of study cohorts

A total of 617 psoriasis patients who initiated IL-17A inhibitor therapy from the SPEECH cohort were finally included. According to a 6:4 random allocation, 370 patients constituted the training cohort and 247 were assigned to the internal validation cohort. To ensure external validity, the study results were tested in a separate cohort of 511 patients with psoriasis treated with IL-17A inhibitor across 26 Chinese hospitals. The baseline characteristics of the three cohorts are summarized in Table 1. The median age was 44 years (IQR, 32–59) in the training cohort, 42 years (IQR, 31–57) in the internal validation cohort, and 39 years (IQR, 31–52) in the external validation cohort. Male patients predominated across all cohorts, accounting for 75.1%, 74.9%, and 69.7% of the populations, respectively. Median BMI values were similar, ranging from 24.3 to 25.0 kg/m^2^. Approximately one-quarter of patients reported a positive family history of psoriasis (22.7%–25.9%). Median baseline PASI scores were consistent across groups, ranging from 13 (IQR, 10–17) to 14 (IQR, 10–21).

**TABLE 1:** Baseline demographic and clinical characteristics of patients in the training, internal validation, and external validation cohorts.

Characteristic	Training cohort (*n* = 370)	Internal validation cohort (*n* = 247)	External validation cohort (*n* = 511)
Age, median (IQR)	44 (32, 59)	42 (31, 57)	39 (31, 52)
Sex, *n* (%)			
Female	92 (24.9%)	62 (25.1%)	155 (30.3%)
Male	278 (75.1%)	185 (74.9%)	356 (69.7%)
BMI, kg/m^2^, median (IQR)	24.7 (22.6, 27.1)	25.0 (22.4, 27.4)	24.3 (22, 26.9)
Family history, *n* (%)			
No	286 (77.3%)	183 (74.1%)	388 (75.9%)
Yes	84 (22.7%)	64 (25.9%)	123 (24.1%)
Smoking, *n* (%)			
No	220 (59.2%)	149 (60.3%)	322 (63.0%)
Yes	150 (40.8%)	98 (39.7%)	189 (37.0%)
Alcohol, *n* (%)			
No	297 (80.3%)	198 (80.2%)	409 (80.0%)
Yes	73 (19.7%)	49 (19.8%)	102 (20.0%)
Prior biologic treatment, *n* (%)			
No	312 (84.3%)	221 (89.5%)	454 (88.8%)
Yes	58 (15.7%)	26 (10.5%)	57 (11.2%)
Education level, *n* (%)			
High school graduate or less	142 (38.6%)	78 (31.7%)	186 (36.4%)
Some college	89 (24.1%)	74 (30.4%)	160 (31.3%)
College graduate or higher	139 (37.3%)	95 (37.9%)	165 (32.3%)
Obese, *n* (%)			
No	282 (76.2%)	188 (76.3%)	362 (70.8%)
Yes	88 (23.8%)	59 (23.7%)	149 (29.2%)
Hypertension, *n* (%)			
No	255 (69.0%)	172 (69.9%)	387 (75.8%)
Yes	115 (31.0%)	75 (30.1%)	124 (24.2%)
Hyperlipidemia, *n* (%)			
No	333 (90.0%)	220 (89.1%)	444 (86.9%)
Yes	37 (10.0%)	27 (10.9%)	67 (13.1%)
Baseline PASI score, median (IQR)	14 (10, 20)	13 (10, 17)	14 (10, 21)

BMI, body mass index; PASI, Psoriasis Area and Severity Index; IQR, interquartile range.

### Comparison of baseline features between primary non-responders and responders

In the training cohort, a total of 86 patients (23.2%) met the definition of primary non-response, defined as failure to achieve PASI75 after 12 weeks of IL-17A inhibitor therapy. Compared with responders, primary non-responders were more frequently male (81.4% vs. 73.2%, *p* = 0.049) and had a higher median BMI (25.8 vs. 24.5 kg/m^2^, *p* < 0.001). A positive family history of psoriasis was less frequent (11.5% vs. 26.4%, *p* = 0.003), whereas prior biologic exposure was more common (26.7% vs. 12.7%, *p* = 0.010). Obesity was also more prevalent among non-responders (30.2% vs. 21.8%, *p* = 0.043). In terms of baseline disease severity, PASI scores were higher in non-responders compared with responders (median 16 vs. 13, *p* = 0.040). Other variables, including smoking, alcohol consumption, hypertension, hyperlipidemia, and educational attainment, did not significantly differ between the two groups (Table 2).

**TABLE 2:** Baseline demographic and clinical characteristics of patients with primary non-response and treatment response in the training cohort.

Characteristic	Total (*n* = 370)	Primary non-response (*n* = 86)	Response (*n* = 284)	*P*-value
Age, median (IQR)	44 (32, 59)	42 (35, 56)	45 (36, 59)	0.430
Sex, *n* (%)				0.049
Female	92 (24.9%)	16 (18.6%)	76 (26.8%)	–
Male	278 (75.1%)	70 (81.4%)	208 (73.2%)	–
BMI, kg/m^2^, median (IQR)	24.7 (22.6, 27.1)	25.8 (24.0, 30.8)	24.5 (22.5, 26.7)	<0.001
Family history, *n* (%)				0.003
No	286 (77.3%)	77 (88.5%)	209 (73.6%)	–
Yes	84 (22.7%)	9 (11.5%)	75 (26.4%)	–
Smoking, *n* (%)				0.974
No	220 (59.2%)	51 (59.3%)	169 (59.5%)	–
Yes	150 (40.8%)	35 (40.7%)	115 (40.5%)	–
Alcohol, *n* (%)				0.195
No	297 (80.3%)	72 (83.9%)	225 (79.2%)	–
Yes	73 (19.7%)	14 (16.1%)	59 (20.8%)	–
Prior biologic treatment, *n* (%)				0.010
No	312 (84.3%)	63 (73.3%)	249 (87.3%)	–
Yes	58 (15.7%)	23 (26.7%)	35 (12.7%)	–
Education level, *n* (%)				0.187
High school graduate or less	142 (38.6%)	36 (42.0%)	106 (32.1%)	–
Some college	89 (24.1%)	22 (25.6%)	67 (23.6%)	–
College graduate or higher	139 (37.3%)	28 (32.4%)	111 (39.1%)	–
Obese, *n* (%)				0.043
No	282 (76.2%)	60 (69.8%)	220 (78.2%)	–
Yes	88 (23.8%)	26 (30.2%)	62 (21.8%)	–
Hypertension, *n* (%)				0.999
No	255 (69.0%)	58 (67.0%)	197 (69.4%)	–
Yes	115 (31.0%)	28 (33.0%)	87 (30.6%)	–
Hyperlipidemia, *n* (%)				0.795
No	333 (90.0%)	77 (89.7%)	256 (90.1%)	–
Yes	37 (10.0%)	9 (10.3%)	28 (9.9%)	–
Baseline PASI score, median (IQR)	14 (10, 20)	16 (11, 23)	13 (10, 19)	0.040

BMI, body mass index; PASI, Psoriasis Area and Severity Index; IQR, interquartile range.

### Baseline clinical predictors of primary non-response

To further explore independent risk factors, logistic regression analyses were conducted (Table 3). Higher BMI (OR 1.13; 95% CI 1.07–1.20), absence of family history of psoriasis (OR 0.35; 95% CI 0.17–0.71), prior biologic treatment (OR 2.19; 95% CI 1.15–4.16), higher baseline PASI score (OR 1.02; 95% CI 1.04–1.09), and male sex (OR 1.21; 95% CI 1.06–1.70) were significantly associated with an increased risk of non-response in the univariable analysis (Table 3). When these variables were further evaluated in multivariable logistic regression, four independent predictors of primary non-response were retained. Higher BMI (OR 1.09; 95% CI 1.02–1.17), family history of psoriasis (OR 0.39; 95% CI 0.18–0.85), prior biologic treatment (OR 2.05; 95% CI 1.01–4.60), and higher baseline PASI score (OR 1.08; 95% CI 1.04–1.12) were identified as independent predictors of treatment outcomes. In contrast, sex was not significantly associated with treatment outcomes in the adjusted analysis (Table 3).

**TABLE 3:** Univariable and multivariable logistic regression analyses of baseline predictors for primary non-response at week 12.

Characteristic	Univariable analysis			Multivariable analysis		
	OR	95% CI	*P*-value	OR	95% CI	*P*-value
Age, median (IQR)	0.99	0.97, 1.01	0.405	1.00	0.97, 1.02	0.772
Sex, n (%)						
Female	–	–	–	–	–	–
Male	1.21	1.06, 1.70	0.031	1.16	0.48, 2.82	0.740
BMI, kg/m^2^, median (IQR)	1.13	1.07, 1.20	<0.001	1.09	1.02, 1.17	0.012
Family history, *n* (%)						
No	–	–	–	–	–	–
Yes	0.35	0.17, 0.71	0.010	0.39	0.18, 0.85	0.017
Smoking, *n* (%)						
No	–	–	–	–	–	–
Yes	0.84	0.47, 1.50	0.553	0.89	0.39, 2.03	0.788
Alcohol, *n* (%)						
No	–	–	–	–	–	–
Yes	0.72	0.35, 1.50	0.383	0.42	0.15, 1.18	0.098
Prior biologic treatment, *n* (%)						
No	–	–	–	–	–	–
Yes	2.19	1.15, 4.16	0.017	2.05	1.01, 4.60	0.042
Education level, *n* (%)						
High school graduate or less	–	–	–	–	–	–
Some college	1.11	0.55, 2.27	0.768	0.90	0.36, 2.21	0.812
College graduate or higher	0.77	0.40, 1.49	0.445	0.72	0.29, 1.78	0.473
Hypertension, *n* (%)						
No	–	–	–	–	–	–
Yes	0.68	0.33, 1.41	0.304	0.65	0.27, 1.56	0.332
Hyperlipidemia, *n* (%)						
No	–	–	–	–	–	–
Yes	0.97	0.39, 2.41	0.954	0.73	0.22, 2.35	0.594
Baseline PASI score, median (IQR)	1.02	1.04, 1.09	0.025	1.08	1.04, 1.12	0.013

BMI, body mass index; PASI, Psoriasis Area and Severity Index; OR, odds ratio; CI, confidence interval; IQR, interquartile range.

### Early treatment response as a predictor of primary non-response

**FIGURE 1:**
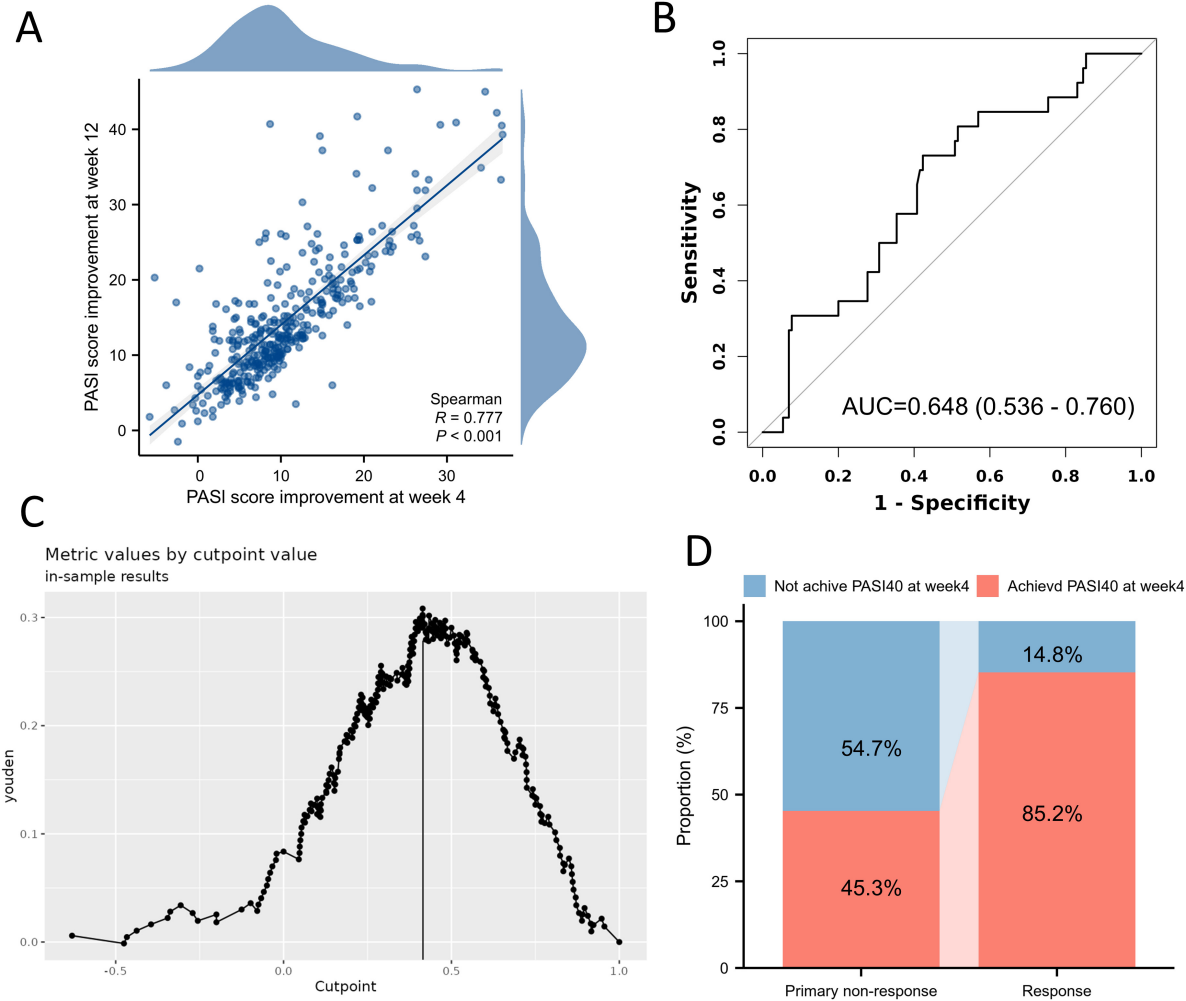
Early treatment response as a predictor of primary non-response to interleukin-17A (IL-17A) inhibitor. **(A)** Correlation between PASI improvement at week 4 and week-12 outcomes. **(B)** Receiver operating characteristic (ROC) analysis for week-4 PASI percentage improvement predicting primary non-response at week 12. **(C)** Identification of the optimal cutoff value based on the Youden index, with PASI40 representing the best threshold for prediction. **(D)** Proportion of patients achieving PASI40 at week 4 stratified by week-12 outcomes. PASI, Psoriasis Area and Severity Index; ROC, receiver operating characteristic.

Previous studies have consistently demonstrated that early clinical response is an important predictor of treatment outcomes in patients receiving biologic therapy (13, 14); therefore, we examined whether early PASI improvement at week 4 predicts primary treatment outcomes at week 12. Week-4 PASI improvement showed a strong correlation with week-12 outcomes (Spearman *r* = 0.777, *p* < 0.001; Figure 1A). ROC analysis identified the optimal cutoff (Figure 1B), with a 40% improvement from baseline (PASI40) at week 4 yielding the highest Youden index and serving as the best predictive threshold (Figure 1C). Patients who ultimately responded at week 12 were more likely to have achieved PASI40 at week 4 than those with primary non-response (85.2% vs. 45.3%) (Figure 1D). These findings support incorporating early response into the prediction framework to enhance risk stratification.

### Development of a multivariable model for predicting primary non-response

**FIGURE 2:**
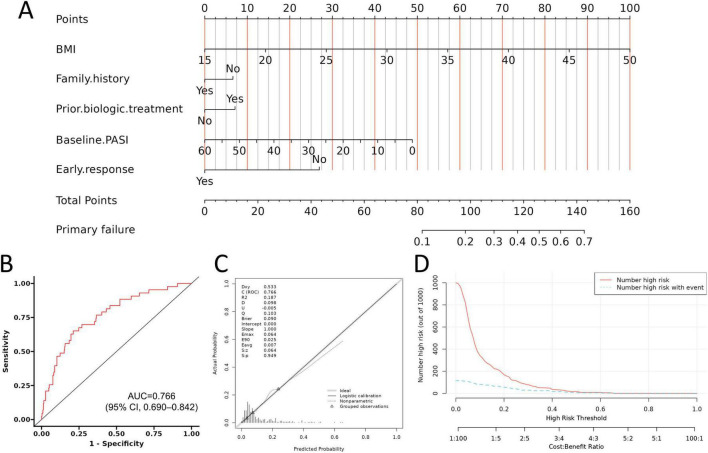
Development and performance evaluation of the multivariable prediction model for primary non-response. **(A)** Construction of the multivariable model incorporating baseline BMI, family history of psoriasis, prior biologic treatment, baseline PASI score, and early PASI40 response. **(B)** ROC curve of the model. **(C)** Calibration plot demonstrating close agreement between predicted and observed probabilities of primary non-response. **(D)** DCA evaluating the clinical utility of the model across a range of threshold probabilities. BMI, body mass index; PASI, Psoriasis Area and Severity Index; ROC, receiver operating characteristic; AUC, area under the curve; DCA, decision curve analysis.

After identifying baseline characteristics and early response as key predictors of primary non-response, we evaluated the performance of the integrated multivariable model (Figure 2A). This model, which incorporated baseline BMI, family history of psoriasis, prior biologic treatment, baseline PASI score, and early PASI40 response, demonstrated good discriminative ability, with an AUC of 0.766 (95% CI, 0.690–0.842) (Figure 2B). The calibration plots showing good concordance between predicted and observed probabilities of non-response (Figure 2C). Quantitatively, the calibration intercept was close to zero, the calibration slope was close to one, and the Brier score was low (Figure 2C), indicating good agreement between predicted and observed risks across the entire probability range. DCA further demonstrated greater net clinical benefit across thresholds of 0–60% compared with “treat-all” or “treat-none” strategies (Figure 2D).

### Internal and external validation of the prediction model

**FIGURE 3:**
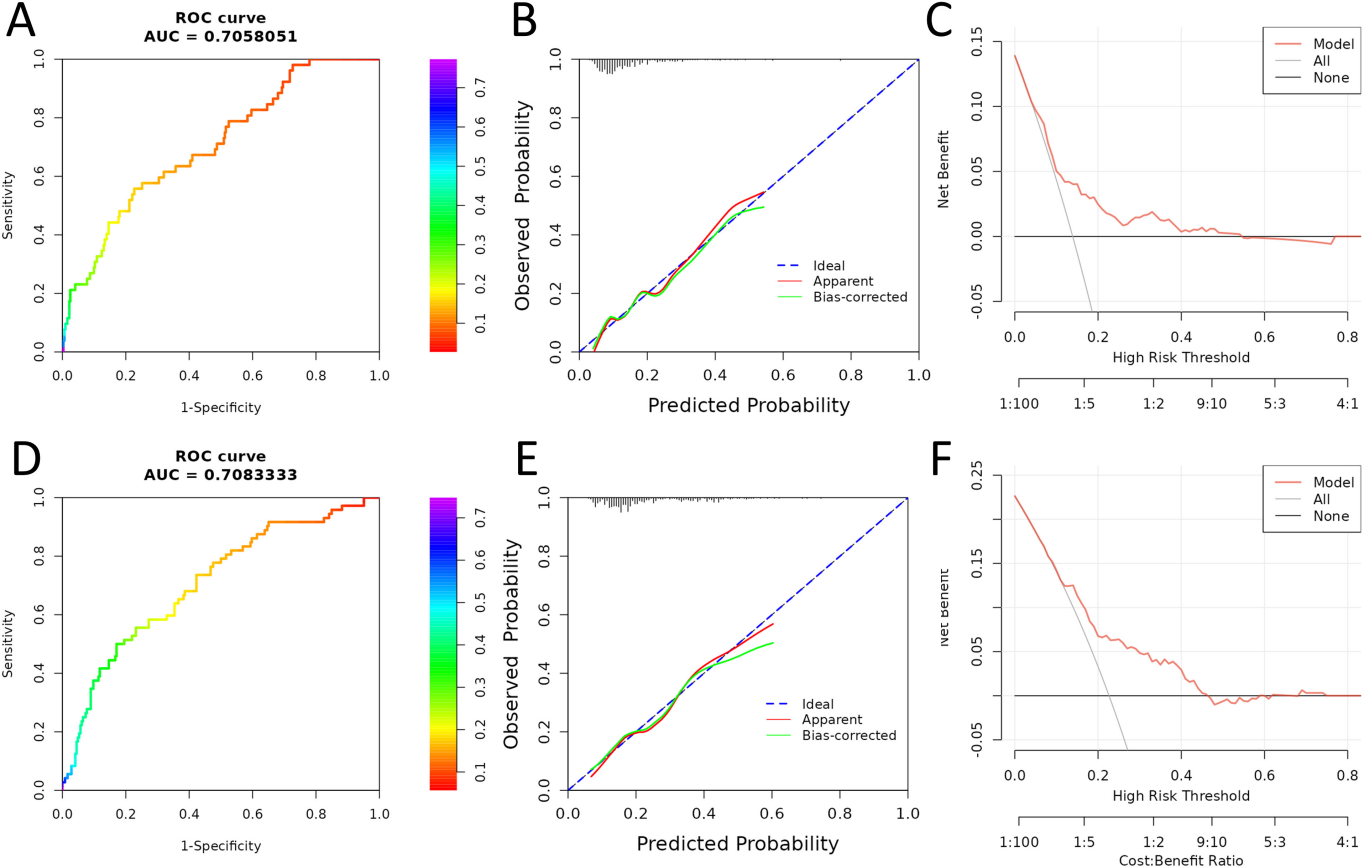
Internal and external validation of the prediction model for primary non-response. **(A–C)** Internal validation cohort: **(A)** ROC curve demonstrating discriminative performance; **(B)** calibration plot showing agreement between predicted and observed probabilities; **(C)** DCA assessing net clinical benefit across a range of threshold probabilities. **(D–F)** External validation cohort: **(D)** ROC curve; **(E)** calibration plot; **(F)** DCA evaluating the clinical utility of the model. BMI, body mass index; PASI, Psoriasis Area and Severity Index; ROC, receiver operating characteristic; AUC, area under the curve; DCA, decision curve analysis.

The performance of the constructed prediction model was further validated in both internal and external cohorts. In the internal validation cohort, the model demonstrated a robust discriminative capacity, with an AUC of 0.706 (Figure 3A). Calibration analysis showed good agreement between predicted and observed probabilities of primary non-response (Figure 3B). Decision curve analysis indicated that the model provided a consistently higher net clinical benefit than either the “treat all” or “treat none” strategies, particularly when the threshold probability was within 0%–60% (Figure 3C). In the independent external validation cohort, the predictive model maintained satisfactory discrimination, achieving an AUC of 0.708 (Figure 3D). Similarly, calibration plots confirmed the accuracy of predicted probabilities (Figure 3E). DCA results again supported the clinical utility of the model (Figure 3F). Together, these findings indicate that the model exhibits good generalizability and robustness across different patient populations, supporting its applicability in real-world clinical settings.

### Development of an online prediction tool

**FIGURE 4:**
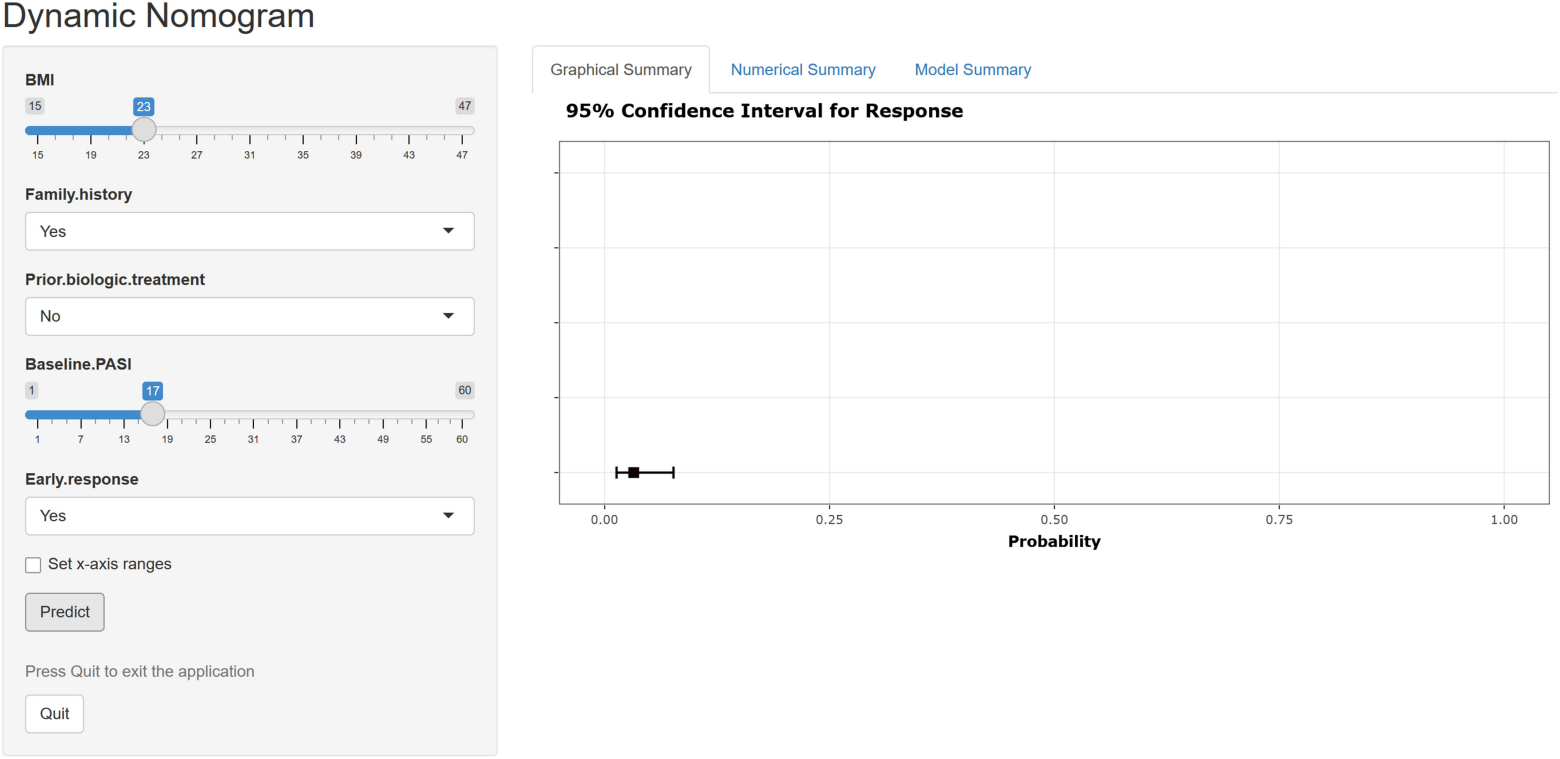
Web-based prediction tool for individualized risk estimation of primary non-response. The figure illustrates an example patient with BMI of 23 kg/m^2^, positive family history of psoriasis, no prior biologic exposure, baseline PASI score of 12, and achievement of PASI40 at week 4, who was classified as having an extremely low risk of primary non-response. BMI, body mass index; PASI, Psoriasis Area and Severity Index.

To facilitate individualized risk estimation in clinical practice, we further developed a web-based prediction tool^1^ integrating the identified predictors. As an illustrative example, the Figure 4 demonstrates a patient with the following profile: BMI of 23 kg/m^2^, positive family history of psoriasis, no prior biologic treatment, baseline PASI score of 12, and achievement of PASI40 at week 4. According to the model, this patient’s probability of experiencing primary non-response was extremely low (Figure 4). This web-based calculator provides a simple, accessible, and clinically relevant platform for real-time risk stratification, allowing physicians to identify high-risk patients early and tailor treatment strategies accordingly.

## Discussion

In this study, we systematically investigated predictors of primary treatment failure to IL-17A inhibitors in psoriasis patients. Using both a training cohort and an independent external validation cohort, we identified several baseline clinical features and early treatment response that were significantly associated with primary non-response. Based on these variables, we constructed and validated a practical prediction model, which was subsequently implemented as an online calculator. The model showed strong discrimination, reliable calibration, and consistent clinical utility across patient groups, supporting its use for individualized risk stratification.

Our analysis revealed that BMI was an important determinant of treatment response. Patients with higher BMI were more likely to experience primary non-response, consistent with earlier studies reporting reduced efficacy of biologics in obese patients, potentially due to altered pharmacokinetics and persistent systemic inflammation (15). Sex-related differences have also been reported, with women showing better PASI responses than men, but these findings were often unadjusted for confounders such as BMI (16). In our study, male sex was linked to poorer response in univariable analysis but lost significance after adjustment, indicating that the apparent sex disparity was largely explained by higher body weight in men rather than sex itself.

Another interesting finding of our study is the role of family history of psoriasis in predicting treatment outcomes with IL-17A inhibitors. In our cohorts, patients with a positive family history were significantly less likely to experience primary non-response (11.5% vs. 26.4%). Consistent with this observation, previous studies have shown that such patients were less likely to discontinue or switch biologic therapy, suggesting a more durable treatment course (17). Moreover, other studies reported that patients with a positive family history were more likely to achieve super-response and to maintain longer drug survival under biologic treatment (18). Future research integrating genetic and clinical data may further clarify the mechanisms by which family history shapes IL-17A inhibitor responses.

We further found that prior biologic exposure negatively impacted treatment outcomes. This observation aligns with previous reports showing that biologic-naïve patients generally respond better to subsequent biologic therapy (19). Mechanistically, repeated or prolonged cytokine blockade may induce immune adaptations that attenuate responsiveness to further targeted interventions (20). Importantly, our study confirmed that early treatment response is a strong predictor of later outcomes. Patients who achieved PASI40 improvement at week 4 were far less likely to experience primary failure at week 12. This is consistent with *post hoc* analyses of phase II/III trials of ixekizumab and secukinumab, where early PASI improvement predicted subsequent PASI75 or PASI90 responses (13). Our results extend these findings by identifying PASI40 as the optimal early threshold for IL-17A inhibitors in routine practice.

These findings have several practical implications. First, clinicians can use baseline characteristics together with early treatment response to identify high-risk patients early in the treatment course. For such patients, closer monitoring or earlier therapeutic adjustments may be warranted to avoid prolonged ineffective therapy. Second, the development of an online prediction tool provides an accessible platform for real-time individualized risk estimation, enhancing shared decision-making between physicians and patients. This is particularly valuable in resource-limited settings, where inappropriate treatment persistence imposes both economic and psychological burdens.

The strengths of our study include the use of two large, independent, multicenter real-world cohorts, which enhances the robustness and generalizability of the findings. Moreover, the integration of both baseline characteristics and early treatment response into a validated predictive model offers a comprehensive approach to risk stratification. Nevertheless, several limitations should be acknowledged. First, as with any observational study, residual confounding and selection bias cannot be entirely ruled out. Second, we did not include laboratory biomarkers or genetic parameters, which may further refine prediction but are less accessible in routine practice. Third, the follow-up period was limited to 12 weeks, and longer-term outcomes such as sustained response or drug survival warrant further investigation. Several clinically relevant disease features, including involvement of difficult-to-treat areas (such as palmoplantar, scalp, and genital psoriasis) and nail disease, were not included in the model because these variables were not uniformly collected across centers. As these features may influence treatment response, future studies with more granular phenotypic data may further refine and enhance the predictive performance of the model.

## Data Availability

The raw data supporting the conclusions of this article will be made available by the authors, without undue reservation.

## Footnotes

1
https://tjspine.shinyapps.io/dynnomapp
